# Interaction among homeodomain transcription factors mediates ethylene biosynthesis during pear fruit ripening

**DOI:** 10.1093/hr/uhae086

**Published:** 2024-03-28

**Authors:** Su-Hao Cao, Zhi-Hua Guo, Hong Liu, Guo-Ming Wang, Kai-Jie Qi, Ze-Wen Wang, Rui-Ping Tian, Shou-Feng Sha, Shao-Ling Zhang, Chao Gu

**Affiliations:** Jiangsu Engineering Research Center for Pear, State Key Laboratory of Crop Genetics and Germplasm Enhancement, Nanjing Agricultural University, Nanjing 210095, China; Jiangsu Engineering Research Center for Pear, State Key Laboratory of Crop Genetics and Germplasm Enhancement, Nanjing Agricultural University, Nanjing 210095, China; Jiangsu Engineering Research Center for Pear, State Key Laboratory of Crop Genetics and Germplasm Enhancement, Nanjing Agricultural University, Nanjing 210095, China; Jiangsu Engineering Research Center for Pear, State Key Laboratory of Crop Genetics and Germplasm Enhancement, Nanjing Agricultural University, Nanjing 210095, China; Jiangsu Engineering Research Center for Pear, State Key Laboratory of Crop Genetics and Germplasm Enhancement, Nanjing Agricultural University, Nanjing 210095, China; Jiangsu Engineering Research Center for Pear, State Key Laboratory of Crop Genetics and Germplasm Enhancement, Nanjing Agricultural University, Nanjing 210095, China; State Key Laboratory of Crop Genetics and Germplasm Enhancement, Nanjing Agricultural University, Nanjing 210095, China; Insitute of Pomology, Liaoning Academy of Agricultural Sciences, Yingkou 115009, China; Jiangsu Engineering Research Center for Pear, State Key Laboratory of Crop Genetics and Germplasm Enhancement, Nanjing Agricultural University, Nanjing 210095, China; Jiangsu Engineering Research Center for Pear, State Key Laboratory of Crop Genetics and Germplasm Enhancement, Nanjing Agricultural University, Nanjing 210095, China

## Abstract

Fruit ripening is manipulated by the plant phytohormone ethylene in climacteric fruits. While the transcription factors (TFs) involved in ethylene biosynthesis and fruit ripening have been extensively studied in tomato, their identification in pear remains limited. In this study, we identified and characterized a HOMEODOMAIN TF, PbHB.G7.2, through transcriptome analysis. PbHB.G7.2 could directly bind to the promoter of the ethylene biosynthetic gene, *1-aminocyclopropane-1-carboxylic acid synthase* (*PbACS1b*), thereby enhancing its activity and resulting in increased ethylene production during pear fruit ripening. Yeast-two-hybrid screening revealed that PbHB.G7.2 interacted with PbHB.G1 and PbHB.G2.1. Notably, these interactions disrupted the transcriptional activation of PbHB.G7.2. Interestingly, PbHB.G1 and PbHB.G2.1 also bind to the *PbACS1b* promoter, albeit different regions from those bound by PbHB.G7.2. Moreover, the regions of PbHB.G1 and PbHB.G2.1 involved in their interaction with PbHB.G7.2 differ from the regions responsible for binding to the *PbACS1b* promoter. Nonetheless, these interactions also disrupt the transcriptional activation of PbHB.G1 and PbHB.G2.1. These findings offer a new mechanism of ethylene biosynthesis during climacteric fruit ripening.

## Introduction

Fruit ripening involves a multitude of metabolic changes that impact various sensory traits such as color, flavor, texture, and aroma. These modifications are not only advantageous for seed dispersal, but also provide essential dietary components, including nutrients and fiber, for both human and animal diets [1–3]. Therefore, elucidating the regulatory mechanisms underlying fruit ripening holds significant importance in enhancing breeding strategies approaches and optimizing cultivation practices, ultimately resulting in improvements in fruit quality.

In climacteric fruits, ripening is characterized by a self-amplifying increase in respiration and ethylene production [4, 5], which is preceded by the biosynthesis of S-adenosyl-L-methionine (SAM) and its conversion to 1-aminocyclopropane-1-carboxylate (ACC) by ACC synthase (ACS), and subsequent conversion of ACC to ethylene by 1-aminocyclopropane-1-carboxylate oxidase (ACO). It is reported that these gene activities are directly regulated by multiple transcription factors (TFs), including Basic helix–loop–helix (bHLH), Apetala2/Ethylene responsive factor (AP2/ERF), Auxin response factor (ARF), MADS-box, NAM/ATAF1/2/CUC2 (NAC), and Homeobox (HB) [6–10]. In these TFs, in apple, MdERF3 induces *MdACS1* transcription to increase ethylene production, and the transcriptional activation of MdERF3 is repressed by the interaction between MdERF3 and MdERF2 [11]. MdERF2 suppresses the transcription of *MdACS1* to impede ethylene biosynthesis, and the transcriptional repression of MdERF2 is disrupted by the interaction between MdERF2 and MdMYC2 (a bHLH TF) [9]. In banana MaERF11 suppresses the transcription of *MaACO1* to negatively affect ethylene biosynthesis during fruit ripening, and this repression is strengthened by the interaction between MaERF11 and MaHDA1 (a histone deacetylase) [12]. Theoretically, TFs involved in the regulation of ethylene biosynthesis may also have interacting partners.

HB TFs constitute a diverse superfamily encompassing various families such as SAWADEE, DDT, KNOX, BELL, WOX, PHD, and HD-ZIP [13, 14]. Multiple studies have demonstrated the involvement of HB TFs in diverse biological processes including leaf development, inflorescence architecture, embryogenesis, asexual reproduction, and fruit replum formation [15–18]. Notably, several HB TFs are also implicated in phytohormone biosynthesis and signal transduction. For instance, in potato, POTH1 decreases gibberellin (GA) accumulation in leaves by suppressing the expression of *GA20-oxidase1* (a key gene for gibberellin biosynthesis) [19]. In rice, OsIPT2 and OsIPT3 decrease GA biosynthesis by suppressing the expression of GA20-oxidase genes, while simultaneously promoting CK biosynthesis by inducing the expression of cytokinin (CK) biosynthesis genes [20]. In *Arabidopsis*, ATHB5 inhibits seed germination and seedling growth in responsive to abscisic acid [21], while ATHB52 negatively modulates primary root elongation by responding to ethylene and impairing auxin distribution and gravitropism [22]. In peach, PpHB.G7 promotes ethylene biosynthesis by enhancing the expression of both *PpACS1* and *PpACO1* [23], whereas tomato LeHB-1 plays a positive role in fruit ripening [24]. These instances indicate that HB TFs may be involved in regulating ethylene biosynthesis and consequently influence fruit ripening processes.

Pear is a widely consumed fruit globally and belongs to the category of climacteric fruit, which exhibit a sharp increase in ethylene production during ripening. To gain insights into the genetic regulation of fruit ripening, in this study, three *HB* genes, namely *PbHB.G7.2*, *PbHB.G1*, and *PbHB.G2.1*, were identified, and their molecular regulation on ethylene biosynthesis was elucidated. These results uncover a new mechanism underlying ethylene biosynthesis in pear fruit.

## Results

### *PbACS1b*, *PbACO1*, and *PbHB.G7.2* are associated with pear fruit ripening

**Figure 1:**
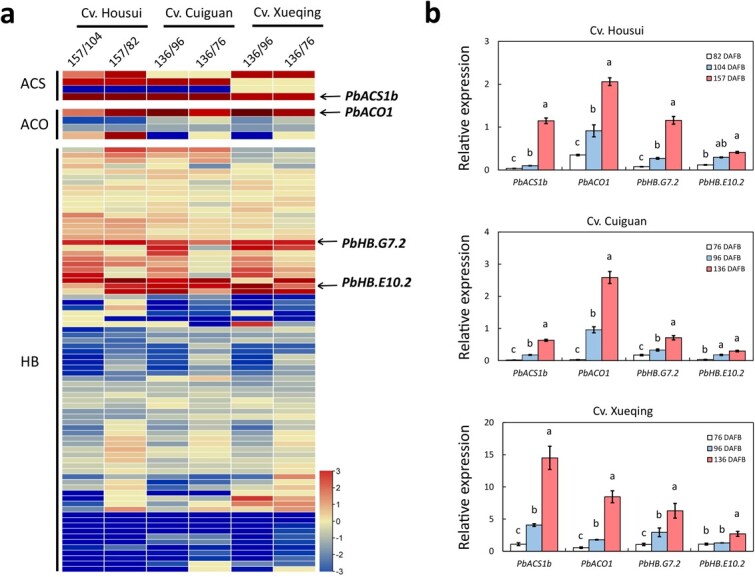
Involvement of *PbACS1b*, *PbACO1*, and *PbHB.G7.2* in pear fruit ripening. **a** Three heatmaps revealed the expression profiles of *ACS*, *ACO*, and *HB* genes between the ripening and enlarging fruits in three pear cvs, Housui, Cuiguan, and Xueqing. **b** Expression analyses of *PbACS1b*, *PbACO1*, *PbHB.G7.2*, and *PbHB.E10.2* in the enlarging and ripening fruits of three pear cultivars: 157 DAFB is the cv. Housui fruit at ripening stage, while 82 and 104 DAFB are the fruits at enlarging stages; 136 DAFB is the cvs. Cuiguan and Xueqing fruit at ripening stage, while 76 and 96 DAB are the fruits at enlarging stages. In cv. Housui, 157/104 represents the fold-change in RPKM value between 157 and 104 DAFB, while 157/82 represents the fold-change in RPKM value between 157 and 82 DAFB. In cvs. Cuiguan and Xueqing, 136/96 represents the fold-change in RPKM value between 136 and 96 DAFB, while 136/76 represents the fold-change in RPKM value between 136 and 76 DAFB.

To identify the *ACS*, *ACO*, and *HB* genes involved in pear fruit ripening, the transcriptome data from cvs. Housui, Cuiguan, and Xueqing fruits, as reported in a previous study [25], were used to perform differential expression analysis of these genes between enlarging and ripening stages (Table S1, see online supplementary material). A total of four *ACS*, four *ACO*, and 78 *HB* genes were detected in the fruit samples from the three pear cultivars (Table S2, see online supplementary material). The detected *ACS* and *ACO* genes were designated based on their orthologs in phylogenetic trees (Fig. S1, see online supplementary material), while the *HB* genes were designated according to a prior study conducted on peach fruit [23]. Among the detected 85 genes, only *PbACS1b*, *PbACO1*, *PbHB.E10.2*, and *PbHB.G7.2* exhibited higher expression levels in ripening fruit compared to enlarging fruit across all three cultivars (Fig. 1a). However, quantitative real-time PCR (qRT-PCR) analysis showed that *PbHB.E10.2* did not display differentially expressed between cv. Housui fruits at 104 and 157 days after flower blooming (DAFB) nor between cv. CG fruits at 96 and 136 DAFB (Fig. 1b). In contrast, *PbACS1b*, *PbACO1*, and *PbHB.G7.2* were more highly expressed in ripening fruit than in enlarging fruit across all three cultivars (Fig. 1b). These results indicate a potential involvement of *PbACS1b*, *PbACO1*, and *PbHB.G7.2* in pear fruit ripening.

### PbHB.G7.2 mediates ethylene biosynthesis in pear fruit callus by inducing the *PbACS1b* expression

**Figure 2:**
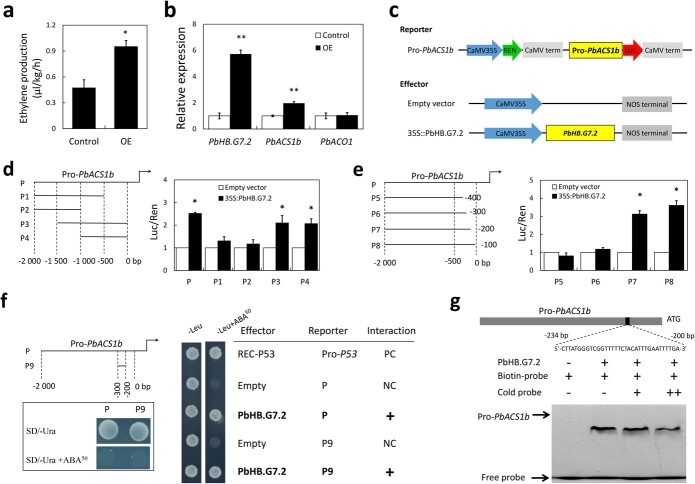
Mediation of PbHB.G7.2 on ethylene biosynthesis and the *PbACS1b* expression in pear fruit callus. **a** Ethylene production was measured in the fruit calli that were either over-expressing PbHB.G7.2 or harboring an empty vector. **b** The qRT-PCR analysis showing the expression levels of *PbHB.G7.2*, *PbACS1b*, and *PbACO1*. **c** The constructed reporters and effectors for the dual-luciferase assay. **d** The *PbACS1*b promoter (P) was divided into four fragments (P1, P2, P3, and P4; Left panel) and then used for the dual-luciferase assay to identify the PbHB.G7.2 binding region (Right panel). **e** The *PbACS1*b promoter (P) was further divided into four additional fragments (P5, P6, P7, and P8; Left panel) and then used for the dual-luciferase assay to narrow the PbHB.G7.2 binding region (Right panel). **f** Reporters harboring the *PbACS1*b promoter (P) or the fragment P9 were screened in yeast cells by 50 ng/mL Aureobasidin A (AbA) (Left panels). The Y1H assay revealed the binding of the fragment P9 by PbHB.G7.2 (Right panel). PC and NC represent positive and negative controls, respectively. **g** A 34-bp fragment within the *PbACS1b* promoter was used as a biotin-labeled probe (Top panel). EMSA showed that PbHB.G7.2 bound to the biotin-labeled probe (Bottom panel). Cold probe concentrations were 10-fold (+) and 100-fold (++) of labeled probes. ‘+’ and ‘–’ indicate the interaction and non-interaction in the Y1H assay and indicate the presence and absence of cold probe, biotin-labeled probe, and recombinant PbHB.G7.2 in the EMSA, respectively.

To test the potential role of PbHB.G7.2 in mediating the expression of *PbACS1b* and *PbACO1*, an over-expression experiment was conducted using pear fruit callus. The result showed that ethylene production was increased in the pear fruit calli over-expressing *PbHB.G7.2*, compared to the fruit calli expressing the empty vector pSAK277 (Fig. 2a). Moreover, *PbHB.G7.2* and *PbACS1b* were more highly expressed in the fruit calli over-expressing *PbHB.G7.2* than in the fruit calli expressing the empty vector pSAK277, while the expression level of *PbACO1* remained relatively unchanged (Fig. 2b). These results indicate that the over-expression of *PbHB.G7.2* promotes ethylene biosynthesis by inducing the expression of *PbACS1b*, while exerting minimal influence on the expression of *PbACO1*.

To test the ability of PbHB.G7.2 to enhance the activity of the *PbACS1b* promoter, 2000-bp sequences upstream of *PbACS1b* were inserted into the pGreenII 0800-LUC vector as a reporter construct (Fig. 2c). The report construct was then infiltrated into tobacco leaves along with the effector construct containing the over-expression vector of *PbHB.G7.2*. Subsequently, the dual-luciferase assay showed that the *LUC* activity driven by the *PbACS1b* promoter (Pro-*PbACS1b*) was significantly increased in the presence of the 35S::PbHB.G7.2 effector construct compared to the empty vector (Fig. 2d), while the *LUC* activity driven by the *PbACO1* promoter was hardly changed (Fig. S2, see online supplementary material). This result suggests that PbHB.G7.2 can enhance the activity of the *PbACS1b* promoter in tobacco leaves.

Because the binding sites of HB TFs remain unknown, to determine the binding region of PbHB.G7.2 within the *PbACS1b* promoter, the promoter was divided into four fragments, P1 (from −2000 bp to −500 bp), P2 (from −2000 bp to −1000 bp), P3 (from −1500 bp to −1 bp), and P4 (from −1000 bp to −1 bp; Fig. 2d), and used as individual reporters in a dual-luciferase assay. The result showed that PbHB.G7.2 enhanced the *LUC* activities driven by either P3 or P4 fragment, while exhibiting no effect on the *LUC* activities driven by either P1 or P2 fragment (Fig. 2d). This result indicates that the PbHB.G7.2 binding region within the *PbACS1b* promoter is likely located within the upstream region spanning from −500 to −1 bp of the initiation codon of *PbACS1b*. Subsequently, the *PbACS1b* promoter was further divided into four additional fragments, P5 (from −2000 bp to −400 bp), P6 (from −2000 bp to −300 bp), P7 (from −2000 bp to −200 bp), and P8 (from −2000 bp to −100 bp; Fig. 2e), and used as individual reporters in a dual-luciferase assay. The result showed that PbHB.G7.2 enhanced the *LUC* activities driven by either P7 or P8 fragments, but while having no impact on the *LUC* activities driven by either P5 or P6 fragments (Fig. 2e). These results provide compelling evidence that the PbHB.G7.2 binding region within the *PbACS1b* promoter is located within the upstream region spanning from −300 bp to −200 bp of the initiation codon of *PbACS1b*.

To test the direct binding capability of PbHB.G7.2 to the *PbACS1b* promoter, the entire sequences (P) and fragment P9 (from −300 bp to −200 bp; Fig. 2f) of the *PbACS1b* promoter were individually inserted into the pAbAi vector. The yeast-one-hybrid (Y1H) assay showed that both the P and P9 fragments of the *PbACS1b* promoter exhibited binding affinity towards PbHB.G7.2 (Fig. 2f), suggesting a potential physical interaction between PbHB.G7.2 and the *PbACS1b* promoter. To validate the Y1H result, a 34-bp fragment within the P9 sequences was synthesized as a biotinylated probe, while recombinant PbHB.G7.2 was generated in *Escherichia coli* (Fig. S3, see online supplementary material). The electrophoretic mobility shift assay (EMSA) showed that the biotinylated probe derived from the *PbACS1b* promoter could be specifically bound by the recombinant PbHB.G7.2, and the strength of the binding signal corresponded inversely with the concentration of the non-labeled competitor probe (cold probe; Fig. 2g). These results definitively demonstrate the physical interaction between PbHB.G7.2 and the *PbACS1b* promoter.

### Pear fruit ripening is regulated by ethylene

**Figure 3:**
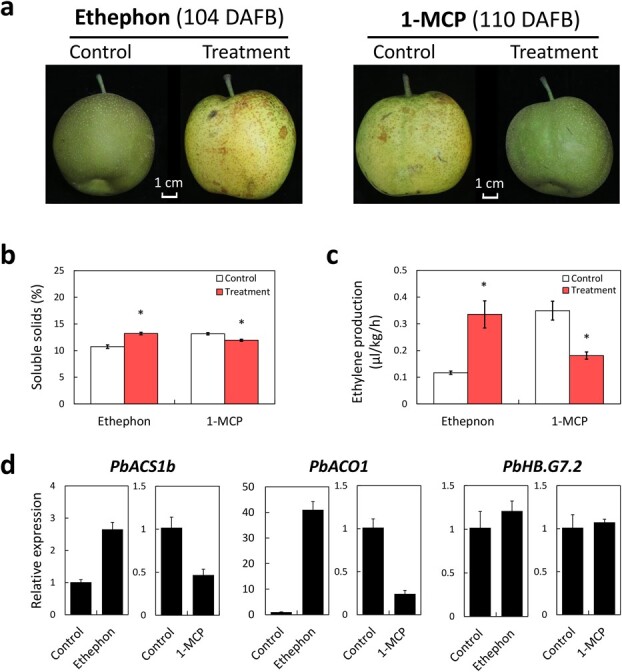
Ethylene positively regulates pear fruit ripening. **a** Left panel shows that the pear fruit was treated by ethephon at 80 DAFB and was harvested at 104 DAFB. Right panel shows that the pear fruit was treated by ethephon at 80 DAFB and was harvested at 110 DAFB. Control and treatment represent untreated and ethephon/1-MCP-treated fruits, respectively. **b** Soluble solids was measured in ethephon−/1-MCP-treated and control fruits. **c** Ethylene production was measured in ethephon−/1-MCP-treated and control fruits. **d** Expression analyses of *PbACS1b*, *PbACO1*, and *PbHB.G7.2* in ethephon−/1-MCP-treated and control fruits.

To test the role of ethylene in the regulation of pear fruit ripening, ethephon and an ethylene inhibitor, 1-methylcyclopropene (1-MCP), were used to treat on-tree fruits of the cv. CG (Fig. 3a). As a result, fruit ripening was delayed by 1-MCP treatment and was accelerated by ethephon treatment. Moreover, soluble solid content and ethylene production decreased in the fruit treated with 1-MCP and increased in the fruit treated with ethephon, compared to the control fruit (Fig. 3b and c). These results are consistent with the positive regulatory effect of ethylene on pear fruit ripening. In addition, the expression of *PbACS1b* and *PbACO1* were down-regulated in the fruit treated with 1-MCP and up-regulated in the fruit treated with ethephon, compared to controls (Fig. 2d). However, the expression of *PbHB.G7.2* remained largely unchanged in response to any treatment or the control condition (Fig. 2d), indicating that ethylene stimulation did not induce the expression of *PbHB.G7.2* in pear fruit.

### PbHB.G7.2 mediates ethylene biosynthesis during pear fruit ripening

**Figure 4:**
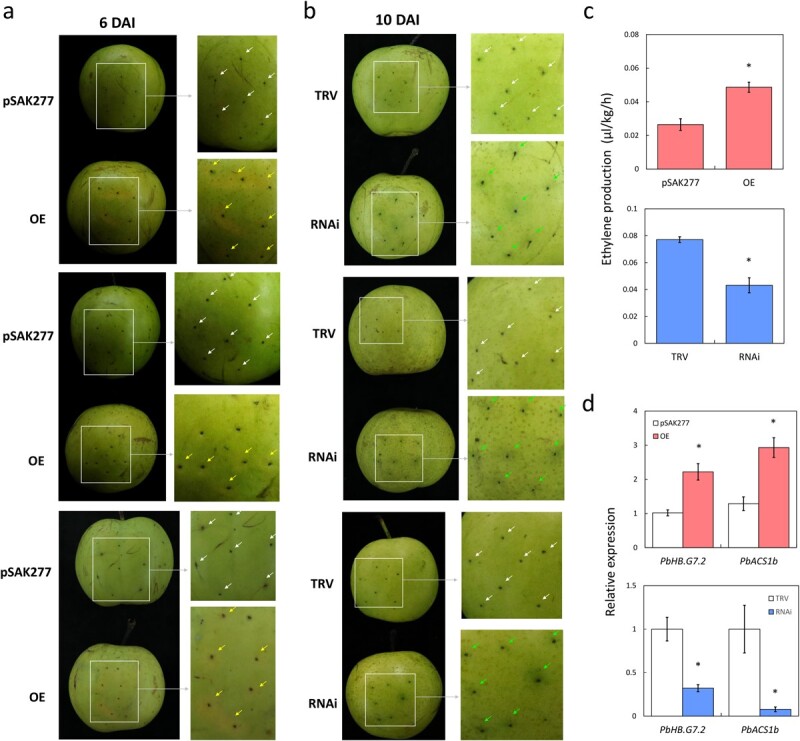
Regulation of PbHB.G7.2 on fruit ripening and ethylene biosynthesis. **a** Transient over-expression of *PbHB.G7.2* in cv. SC1 fruits accelerated fruit ripening. DAI represents days after infiltration. **b** Transient silencing of *PbHB.G7.2* in cv. Sucui No.1 fruits delayed fruit ripening. **c** Ethylene production was measured in the transient transformed fruits. **d** Expression analyses of *PbACS1b* and *PbHB.G7.2* in the fruits over-expressing (Top panel) or silencing *PbHB.G7.2* (Bottom panel). OE and pSAK277 represent over-expression of *PbHB.G7.2* and the empty vector pSAK277, respectively. RNAi and TRV represent silencing of *PbHB.G7.2* and the empty vectors pTRV1 and pTRV2, respectively. Arrows represent the positions of cv. Sucui No.1 fruits infiltrated by *Agrobacterium*.

To clarify the function of PbHB.G7.2 in ethylene biosynthesis and fruit ripening, transient over-expression and silencing experiments were performed on cv. Sucui No.1 fruit. As a result, the pericarp of the fruit over-expressing *PbHB.G7.2* presented a yellow color, in contrast to the fruit expressing the empty vector pSAK277 (Fig. 4a). Conversely, the pericarp of the fruit with silenced *PbHB.G7.2* presented a green color compared to the fruit expressing the empty vectors pTRV1 and pTRV2 (TRV; Fig. 4b). This result suggests that PbHB.G7.2 positively contributes to pear fruit ripening. Moreover, ethylene production increased in the fruit over-expressing *PbHB.G7.2*, while it decreased in the fruit with silenced *PbHB.G7.2*, compared to the controls (Fig. 4c). The result suggests that PbHB.G7.2 has a positive effect on ethylene biosynthesis. In addition, the expression levels of *PbACS1b* and *PbHB.G7.2* were up-regulated in the fruit over-expressing *PbHB.G7.2*, whereas they were down-regulated in the fruit with silenced *PbHB.G7.2*, in comparison to the control fruit (Fig. 4d). These results suggest that PbHB.G7.2 positively affects ethylene biosynthesis by inducing the expression of *PbACS1b*.

### PbHB.G7.2 interacts with PbHB.G1 and PbHB.G2.1

**Figure 5:**
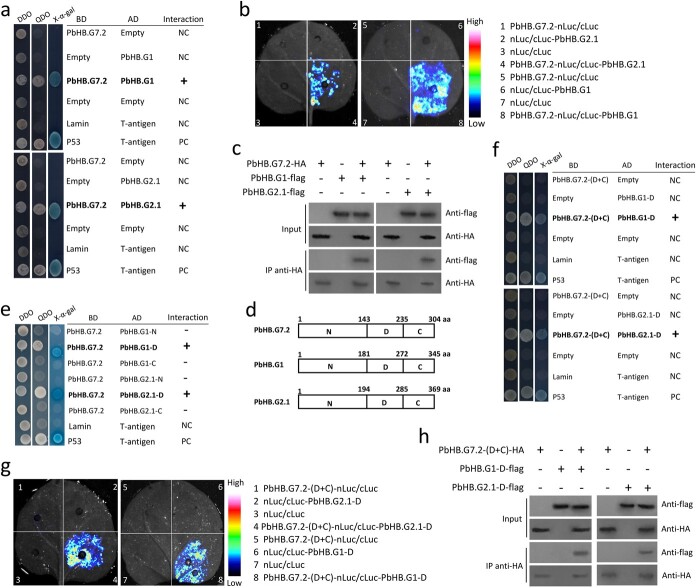
Physical interaction between PbHB.G7.2 and PbHB.G1 as well as PbHB.G2.1. The interaction was tested by the Y2H (**a**), FLCI (**b**), and Co-IP (**c**). **d** PbHB.G7.2, PbHB.G1, and PbHB.G2.1 were divided into three fragments. aa represents amino acid. **e** The Y2H assay showed that PbHB.G7.2 interacts with PbHB.G1-D and PbHB.G2.1-D. The interaction between PbHB.G7.2-(D + C) and PbHB.G1-D or PbHB.G2.1-D was tested by Y2H (**f**), FLCI (**g**), and Co-IP (**h**). DDO, SD medium lacking Trp and Leu; QDO, SD medium lacking Trp, Leu, His, and Ade; X-a-gal, QDO medium containing x-a-gal and AbA. ‘+’ and ‘–’ indicate the interaction and non-interaction in the Y2H assays and indicate the presence and absence of PbHB.G7.2-HA, PbHB.G1-flag, PbHB.G2.1-flag, PbHB.G7.2-(D + C)-HA, PbHB.G1-D-flag, or PbHB.G2.1-D-flag fusion proteins in the Co-IP assays, respectively.

To identify proteins that interact with PbHB.G7.2, a yeast-two-hybrid (Y2H) screening was performed using a cv. Cuiguan fruit cDNA library. Ten proteins were identified from the screening, using PbHB.G7.2 as bait (Table S3, see online supplementary material). The Y2H assay showed that of these proteins, PbHB.G1 (Pbr035813.1) and PbHB.G2.1 (Pbr004979.1), which were respectively classified into the clusters G1 and G2 [23], interacted with PbHB.G7.2 (Fig. 5a), while no interaction was observed between PbHB.G7.2 and any other protein (Fig. S4a, see online supplementary material). To validate the Y2H results, further experiments including firefly luciferase complementation imaging (FLCI) and co-immunoprecipitation (Co-IP) assays were performed in tobacco leaves. FLCI showed the presence of Luc fluorescence only in tobacco leaves co-infiltrated with PbHB.G7.2-nLuc along with either cLuc-PbHB.G1 or cLuc-PbHB.G2.1, but not in any control (Fig. 5b). Moreover, PbHB.G1-flag and PbHB.G2-flag co-immunoprecipitated with PbHB.G7.2-HA (Fig. 5c). These results suggest that both PbHB.G1 and PbHB.G2 interact with PbHB.G7.2.

To identify the interaction regions between PbHB.G7.2 and PbHB.G1 as well as PbHB.G2.1, the sequences of these three genes were divided into three fragments: N, D (containing the HOMEOBOX domain), and C regions (Fig. 5d). These protein fragments had no self-activation in yeast cells (Fig. S4b and c). The Y2H assay showed that PbHB.G7.2 interacted with fragment D of both PbHB.G1 and PbHB.G2.1 (PbHB.G1-D and PbHB.G2.1-D), while no interaction was observed with any other fragments of PbHB.G1 and PbHB.G2.1 (Fig. 5e). Further analyses showed that both PbHB.G1-D and PbHB.G2.1-D interacted with the combined fragments D and C of PbHB.G7.2 (PbHB.G7.2-(D + C); Fig. 5f), but not with any other fragment(s) of PbHB.G7.2 (Fig. S4d–h). To confirm this result, FLCI and Co-IP assays were also performed. The FLCI results showed that Luc fluorescence was specifically detected in tobacco leaves co-infiltrated with PbHB.G7.2-(D + C)-nLuc and either cLuc-PbHB.G1-D or cLuc-PbHB.G2.1-D, but not in any control (Fig. 5g). Moreover, PbHB.G1-D-flag and PbHB.G2-D-flag co-immunoprecipitated with PbHB.G7.2-(D + C)-HA (Fig. 5h). These results clearly indicate that fragment D of both PbHB.G1 and PbHB.G2.1 interacts with the combined fragments D and C of PbHB.G7.2.

### The interactions between PbHB.G7.2 and PbHB.G1 or PbHB.G2 disrupt the transcriptional activation of the three HB TFs

**Figure 6:**
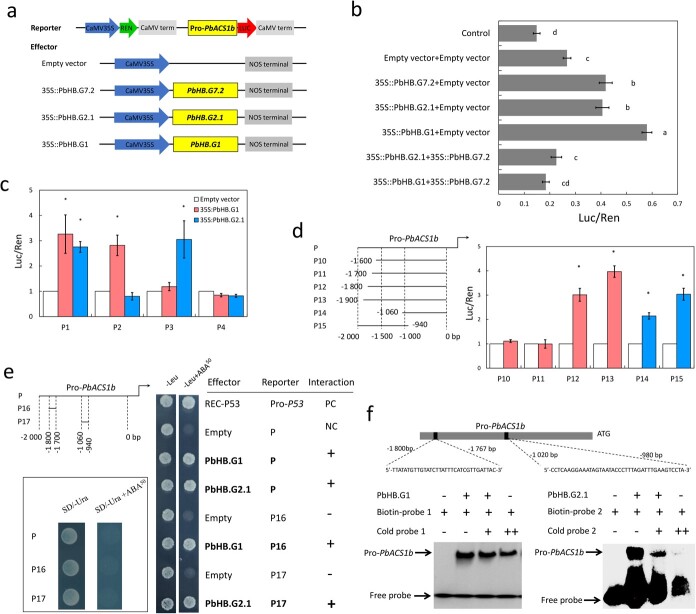
Impact of PbHB.G7.2-PbHB.G1 and PbHB.G7.2-PbHB.G2.1 interactions on the binding of three HB TFs to the *PbACS1b* promoter. **a** The constructed reporter and effectors for the dual-luciferase assay. **b** The dual-luciferase assay showed that the *LUC* activity driven by the *PbACS1b* promoter in leaves infiltrated with 35S::PbHB.G7.2, 35S::PbHB.G1, or 35S::PbHB.G2.1 was stronger than leaves infiltrated with empty vector or co-infiltrated with 35S::PbHB.G7.2 and 35S::PbHB.G1 or 35S::PbHB.G2.1. **c** The dual-luciferase assay showed that the *LUC* activity driven by either P1 or P2 fragments was significantly increased by 35S::PbHB.G1 compared to the empty vector. Similarly, the *LUC* activity driven by either P1 or P3 fragments was significantly increased by 35S::PbHB.G2.1 compared to the empty vector. **d** The *PbACS1*b promoter (P) was divided into six fragments (P10, P11, P12, P13, P14, and P15; Left panel) and then used for the dual-luciferase assay to refine the binding regions of PbHB.G1 and PbHB.G2.1 (Right panel). **e** Reporters harboring the *PbACS1*b promoter (P), the fragments P16, or P17 were screened in yeast cells by 50 ng/mL Aureobasidin A (AbA) (Left panels). The Y1H assay revealed that the fragments P16 and P17 were bound by PbHB.G1 and PbHB.G2.1 (Right panel). PC and NC represent positive and negative controls, respectively. **f** Two fragments in the *PbACS1b* promoter were used as the biotin-labeled probes 1 and 2 (Top panel). EMSA showed that both PbHB.G1 and PbHB.G2.1 bound to the biotin-labeled probes 1 and 2, respectively (Bottom panel). Cold probe concentrations were 10-fold (+) and 100-fold (++) of labeled probes. ‘+’ and ‘–’ indicate the interaction and non-interaction in the Y1H assay and indicate the presence and absence of cold probe, biotin-labeled probe, and recombinant PbHB.G1/PbHB.G2.1 in the EMSA, respectively.

To examine the impact of PbHB.G7.2-PbHB.G1 and PbHB.G7.2-PbHB.G2.1 interactions on the transcriptional activation of PbHB.G7.2, the coding sequences of *PbHB.G1* and *PbHB.G2.1* were inserted into the pSAK277 vector as effector constructs (Fig. 6a). The result showed that the *LUC* activity driven by the *PbACS1b* promoter was significantly reduced in leaves over-expressing *PbHB.G7.2* together with either *PbHB.G1* or *PbHB.G2.1*, compared to leaves solely over-expressing *PbHB.G7.2* (Fig. 6b). This result suggests that the interactions of PbHB.G7.2 with PbHB.G1 or PbHB.G2 repress the transcriptional activation of PbHB.G7.2. Interestingly, the activities driven by the *PbACS1b* promoter were higher in leaves over-expressing *PbHB.G1* or *PbHB.G2.1* alone, relative to leaves infiltrated with the empty vector or over-expressing *PbHB.G7.2* together with *PbHB.G1* or *PbHB.G2.1* (Fig. 6b). These results suggest that both PbHB.G1 and PbHB.G2.1 can also enhance the activity of the *PbACS1b* promoter, but their transcriptional activations are also repressed by the interaction with PbHB.G7.2.

### Both PbHB.G1 and PbHB.G2.1 physically interact with the *PbACS1b* promoter

To identify the binding regions of PbHB.G1 and PbHB.G2.1 within the *PbACS1b* promoter, a dual-luciferase assay was performed using fragments P1, P2, P3, and P4 of the *PbACS1b* promoter in conjunction with PbHB.G1 and PbHB.G2.1 constructs. The result showed that PbHB.G1 enhanced the *LUC* activity driven by either P1 or P2 fragments (Fig. 6c), indicating that the binding region of PbHB.G1 within the *PbACS1b* promoter is located within the upstream region from −2000 bp to −1000 bp of the initiation codon of *PbACS1b*. PbHB.G2.1 enhanced the *LUC* activity driven by either P1 or P3 fragments (Fig. 6c), indicating that the binding region of PbHB.G2.1 within the *PbACS1b* promoter likely positioned at the boundary between fragments P2 and P3.

To refine the identification of interaction regions, the *PbACS1b* promoter was further divided into additional fragments P10 (from −1600 bp to −1 bp), P11 (from −1700 bp to −1 bp), P12 (from −1800 bp to −1 bp), P13 (from −1900 bp to −1 bp), P14 (from −1060 bp to −1 bp), and P15 (from −2000 bp to −940 bp), and used for dual-luciferase assay (Fig. 6d). The result showed that PbHB.G1 enhanced the *LUC* activity driven by either P12 or P13 fragments, indicating that the binding region of PbHB.G1 within the *PbACS1b* promoter is located within the upstream region from −1800 bp to −1700 bp of the initiation codon of *PbACS1b*. Moreover, PbHB.G2 enhanced the *LUC* activity driven by P14 and P15 fragments (Fig. 6d), indicating that the binding region of PbHB.G2.1-Pro-*PbACS1b* within the *PbACS1b* promoter is located within the upstream region from −1060 bp to −940 bp of the initiation codon of *PbACS1b*.

To test whether PbHB.G1 and PbHB.G2.1 directly bind to the *PbACS1b* promoter, fragments P16 (from −1800 bp to −1700 bp) and P17 (from −1060 bp to −940 bp; Fig. 6e) were employed in a Y1H assay. The result showed that PbHB.G1 exhibited binding affinity towards both the P16 and P sequences of the *PbACS1b* promoter, while PbHB.G2.1 displayed binding affinity towards both the P17 and P sequences of the *PbACS1b* promoter (Fig. 6e). This result suggests that PbHB.G1 and PbHB.G2.1 may physically bind to the *PbACS1b* promoter.

To confirm the Y1H result, 33-bp and 40-bp sequences from fragments P16 and P17, respectively, were synthesized as biotinylated probes. Additionally, PbHB.G1 and PbHB.G2.1 were recombined in *E. coli* (Fig. S3, see online supplementary material), and the recombinants were used in an EMSA. The results showed that the recombinants of PbHB.G1 and PbHB.G2.1 could effectively bind to the respective biotinylated probes derived from the *PbACS1b* promoter, and the binding signals were observed to be inversely correlated to the concentration of the cold probe (Fig. 6f). Consequently, these findings provide further evidence that both PbHB.G1 and PbHB.G2.1 physically bind to the *PbACS1b* promoter.

### *PbHB.G1* and *PbHB.G2.1* were lesser expressed in ripening fruit than *PbHB.G7.2*

**Figure 7:**
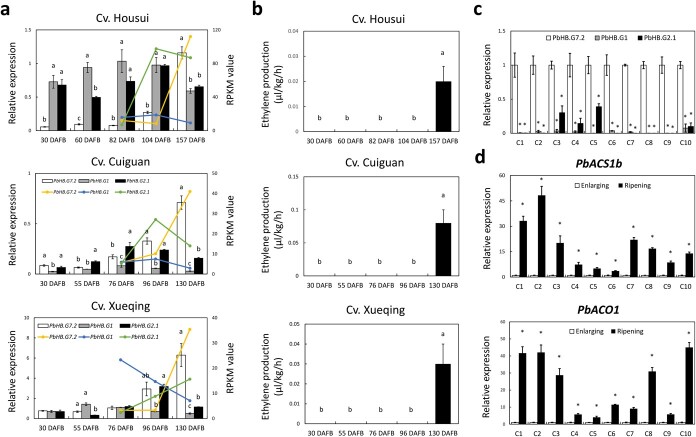
Expression analysis of *PbHB.G7.2*, *PbHB.G1*, and *PbHB.G2.1* in enlarging and ripening fruits. **a** Expression analyses of *PbHB.G7.2*, *PbHB.G1*, and *PbHB.G2.1* in enlarging and ripening fruits of cvs. Housui, Cuiguan, and Xueqing. 30, 60, 82, and 104 DAFB are the fruits at enlarging stages, while 157 DAFB is the fruit at ripening stage in cv. Housui. 30, 55, 76, and 96 DAB are the fruits at enlarging stages, while 136 DAFB is the fruit at ripening stage in cvs. Cuiguan and Xueqing. Each of the colored lines represents the changes in RPKM value, while column charts were calculated by qRT-PCR. **b** Measurements of ethylene production in developing and ripening fruits of three pear cultivars. **c** Expression analyses of *PbHB.G7.2*, *PbHB.G1*, and *PbHB.G2.1* in ripening fruits across 10 other cultivars. **d** Expression analyses of *PbACS1b* (top panel) and *PbACO1* (bottom panel) in enlarging and ripening fruits of 10 additional cultivars. C1 to C10 represents the pear cultivars Huasu, Zhongli No.1, Xizilv, Zaomeisu, Liuyueshuang, Jinhua, Eli No.1, Ningmenghuang, Jinsui No.1, and Xinhang, respectively.

The presence of PbHB.G7.2, PbHB.G1, and PbHB.G2.1 was found to enhance the activity of the *PbACS1b* promoter, and their transcriptional activations were repressed by the interactions of PbHB.G7.2 with PbHB.G1 and PbHB.G2.1. It has confused the specific HB gene(s) responsible for playing a more important role in the *PbACS1b* transcription and ethylene biosynthesis. For this reason, expression levels of *PbHB.G7.2*, *PbHB.G1*, and *PbHB.G2.1* were examined in enlarging and ripening fruits of cvs. Housui, Cuiguan, and Xueqing. The result showed that *PbHB.G7.2* had higher levels of expression than both *PbHB.G1* and *PbHB.G2.1* in ripening fruits, but not in enlarging fruits, across all three pear cultivars (Fig. 7a). Simultaneously, ethylene production was significantly increased in ripening fruits compared to developing fruits in all three cultivars (Fig. 7b). Moreover, to test the prevalence of the expression profile of the three *HB* genes in ripening fruits across various cultivars, ripening fruits from 10 additional cultivars were collected for the qRT-PCR analysis. The result showed that *PbHB.G7.2* had higher levels of expression than *PbHB.G1* and *PbHB.G2.1* in all 10 cultivars (Fig. 7c). These results suggest a more significant role of PbHB.G7.2 in the transcriptional regulation of *PbACS1b* compared to PbHB.G1 and PbHB.G2.1. In addition, to gain further insight into the involvement of *PbACS1b*, *PbACO1*, and *PbHB.G7.2* in fruit ripening, enlarging fruits from 10 additional cultivars were analysed by qRT-PCR. The result showed that *PbACS1b*, *PbACO1*, and *PbHB.G7.2* were more highly expressed in ripening fruits compared to enlarging fruits across all 10 cultivars (Fig. 7d; Fig. S5, see online supplementary material), further supporting their association with pear fruit ripening.

## Discussion

### PbHB.G7.2 mediates ethylene biosynthesis in pear fruit by inducing the *PbACS1b* expression

The ripening mechanism in climacteric fruits has been widely studied in apple [9–11, 26], banana [12, 27, 28], peach [23, 29], and kiwifruit [30, 31]. In contrast, the understanding of genes involved in ethylene biosynthesis and signaling transduction in pear fruit is limited [32–34]. To explore the ripening mechanism in pear fruit, we treated pear fruits (while still on the tree) with exogenous ethephon and 1-MCP and confirmed the positive mediation of ethylene on pear fruit ripening (Fig. 2a). This result is supported by previous reports that demonstrated a delay in pear fruit ripening with 1-MCP treatment [32]. Coincidently, two ethylene biosynthetic genes, *PbACS1b* and *PbACO1*, are positively correlated with fruit ripening and ethylene production (Fig. 2d), suggesting the involvement of both *PbACS1b* and *PbACO1* in ethylene biosynthesis during fruit ripening. Of these two genes, *PbACO1*, an ortholog of apple *MdACO1* (Fig. S1b, see online supplementary material), is identical to the temporarily designated *PbACO54* in a previous study [32]. *PbACS1b*, an ortholog of peach *PpACS1* (Fig. S1a), differs from the previously reported *PuACS1a* in *Pyrus ussuriensis* [33]. The identification of these two genes lays the groundwork for future research on the transcriptional regulation of ethylene biosynthesis during pear fruit ripening.

In perennial fruit trees, ethylene biosynthesis genes are regulated by PpHB.G7 and PpERF.A16 in peach fruit [23, 35]; MdERF3, MdARF5, and MdMYC2 in apple fruit [9–11]; and AdNAC2 and AdNAC6/7 in kiwifruit [30, 36]. However, only PbERF24 has been reported to regulate the expression of *PbACO1* in pear fruit [32]. In this study, we found that PbHB.G7.2 promotes ethylene production during pear fruit ripening by inducing the expression of *PbACS1b* (Figs 3 and 4). Interestingly, PbHB.G7.2 belongs to the HD-ZIP II family and is an ortholog of PpHB.G7, which is known to be involved in peach fruit ripening [23]. This result indicates that the role of HB TFs in group G7 in ethylene biosynthesis during fruit ripening is similar across different fruit trees. The only distinction lies in the fact that PpHB.G7 can bind to both *ACS* and *ACO* promoters, whereas PbHB.G7.2 can only bind to the *ACS* promoter.

### Transcription activation of PbHB.G7.2 is weakened by its interaction with PbHB.G1 and PbHB.G2.1

Increasing evidence has shown the influence of the interacting partner on the transcriptional activation or repression of the TFs involved in fruit ripening [10–12, 28]. The detected protein–protein interactions can either enhance or weaken the transcriptional impact of TFs on the expression of downstream genes. Within the HB family of TFs, interactions have been observed between KNOX proteins and BELL proteins [37, 38]. In this study, we found that PbHB.G1 and PbHB.G2.1 interact with PbHB.G7.2 (Fig. 5). These two HB TFs also belong to the HD-ZIP II family [23] and, like PbHB.G7.2, directly interact with the *PbACS1b* promoter to enhance its activity (Fig. 7). Interestingly, the transcriptional activation of these three HB TFs was disrupted by the interactions of PbHB.G7.2 with PbHB.G1 and PbHB.G2.1 (Fig. 6b). This is similar to a previous report where protein–protein interactions between TFs weakened their transcriptional effect on the expression of downstream genes. For example, the interaction between MdERF3 and MdERF2 repressed the transcriptional activation of MdERF3 [11], while the interaction of MdERF2 with MdMYC2 repressed the transcriptional activation of MdERF2 [9]. Aux/IAA TFs can also repress the transcriptional activation of ARF TFs through protein–protein interactions involving their C-terminal domains [39, 40].

In addition to the detected ERF and HB TFs, protein–protein interactions between two members were also reported in the NAC [41, 42], MADS-box [43, 44], bHLH [45], and WRKY [46] families. However, little is known about the temporal features of these TFs when expressed in tissue. The transcriptional activations of PbHB.G7.2, PbHB.G1, and PbHB.G2.1 on the *PbACS1b* promoter contradict their protein–protein interactions in the present study, making it uncertain which HB TF plays a crucial role in ethylene biosynthesis and fruit ripening. To address this, the expression levels of *PbHB.G7.2*, *PbHB.G1*, and *PbHB.G2.1* were analysed in pear fruits. We found that *PbHB.G1* and *PbHB.G2.1* were expressed at a lower level in ripening fruits compared to *PbHB.G7.2* (Fig. 7). This result suggests that PbHB.G7.2 may have a more important role in ethylene biosynthesis than PbHB.G1 and PbHB.G2.1. Moreover, the lesser expression of *PbHB.G1* and *PbHB.G2.1* in ripening fruit could weaken the interactions of PbHB.G7.2 with PbHB.G1 and PbHB.G2.1, thereby enhancing the transcriptional activation of PbHB.G7.2 on the expression of *PbACS1b* and increasing ethylene production during pear fruit ripening.

## Conclusion

Our result indicates that the identified PbHB.G7.2 positively affects ethylene biosynthesis by inducing the expression of *PbACS1b*, independent of ethylene signaling. However, in enlarging fruits, the low expression of PbHB.G7.2 results in its interaction solely with PbHB.G1 and PbHB.G2.1, leading to a decrease in ethylene production. In ripening fruit, the high expression of PbHB.G7.2 allows for its interaction with PbHB.G1 and PbHB.G2.1, enabling binding to the *PbACS1b* promoter and subsequently increasing ethylene production. Both PbHB.G1 and PbHB.G2.1 are unresponsive to ethylene signal (Fig. S6, see online supplementary material) yet exhibit lower expression levels compared to *PbHB.G7.2* in ripening fruit. These findings differ from previous reports on the molecular regulation of ethylene biosynthesis during fruit ripening.

## Materials and methods

### Plant materials

A total of 13 pear cultivars were maintained at Jiangpu orchard, Nanjing Agricultural University (Nanjing, Jiangsu Province, China). Based on the previous study [25], the fruits of pear cultivars Cuiguang and Xueqing were collected at 30, 55, 76, 96, and 130 DAFB, while the cv. Housui fruits were collected at 30, 60, 82, 104, and 157 DAFB. Moreover, to enhance the reliability of these candidate genes, the ripening fruit of the other 10 pear cultivars, Huasu, Zhongli No.1, Xizilv, Zaomeisu, Liuyueshuang, Jinhua, Eli No.1, Ningmenghuang, Jinsui No.1, and Xinhang, were respectively collected at 140, 142, 145, 145, 128, 156, 150, 152, 142, and 145 DAFB, while the enlarging fruits of these 10 pear cultivars were collected at 65 DAFB. At least four fruits at any stage were included in each of three biological replicates. The flesh sampling regime was identical to a previous report [32].

### Exogenous treatments of ethephon and 1-methylcyclopropene

Exogenous treatments were performed in cv. Cuiguan fruit at approximately 80 DAFB. At least 40 fruits on the tree were soaked in 300 μL l^−1^ ethephon or 1.5 μL l^−1^ 1-methylcyclopropene (1-MCP) solutions for 5 min. To block light and limit gas exchange, the treated fruits were bagged with black airtight papers that were removed after 24 h. The fruits treated with ethephon were collected at the ripening stage, while the fruits treated with 1-MCP were collected on the same day as the control fruits at the ripening stage. At least six fruits were included in each of six biological replicates. The sampling regime was identical to a previous report [32].

### Measurements of soluble solids and ethylene production

Soluble solids were measured as described in a previous study [32]. Ethylene production was measured using a gas chromatographic system (GC2010, Shimadzu, Japan) with a 1000-μL Rheodyne injector (Gaoge, Shanghai, China) according to a previous report [47]. In brief, the fruits in each replicate were weighed and sealed in a 3-L airtight chamber. A 0.5-mL gas sample was collected from the chamber by a 0.5-mL syringe and then injected into a gas chromatograph fitted with a 30-cm glass column (3.2 mm ID).

### Sequence and phylogenetic analyses of *ACS* and *ACO* genes

Pear *ACS* and *ACO* genes were identified from pear (*Pyrus bretschneideri*; http://peargenome.njau.edu.cn/), apple, peach, strawberry, orange, grape, and papaya (https://phytozome.jgi.doe.gov/) genomes, based on the homologies of peach *PpACS1* and *PpACO1* genes [23]. The amino acid sequences were aligned and then used to construct phylogenetic trees as described in a previous report [35].

### Transformation in pear fruit

The fruit of cv. Sucui No.1, which has yellow skin at ripening stage [48], was used for transient transformation. The over-expression vector of *PbHB.G7.2* was constructed by inserting the full-length coding sequences of *PbHB.G7.2* into the pSAK277 vector.

The constructed vector was transformed into *Agrobacterium tumefaciens* strain EHA105 by electroporation. The incubation and injection conditions were identical to a previous report [32]. *Agrobacteria* containing the construct or an empty vector were infiltrated into 40 fruits.

For gene silencing, a 520-bp fragment of *PbHB.G7.2* was inserted into the pTRV2 vector, and then introduced into *A. tumefaciens* strain GV3101. All incubations and transient transformation conditions were identical to a previous report [35]. *Agrobacteria* containing pTRV1 and the construct were co-infiltrated into 40 fruits, while *Agrobacteria* containing the pTRV1 and pTRV2 empty vectors were co-infiltrated into the other 40 fruits. At least five fruits were weighted and sealed in a 3-L airtight jar, and four replicates were prepared. Ethylene production was measured when the phenotype of the fruit over-expressing or silencing *PbHB.G7.2* was different from the control. Each sample of fruit flesh covered by bacteria was collected for qRT-PCR analysis.

Moreover, the *Agrobacterium* harboring the over-expression vector of *PbHB.G7.2* or the empty vector pSAK277 was used to infect the fruit calli of ‘Clapp’s Favorite’ as shown in a previous report [49]. All of the primer sequences are listed in Table S4 (see online supplementary material).

### Dual-luciferase assay

A 2581-bp sequence upstream of *PbACO1*, a 2000-bp sequence upstream of *PbACS1b*, P1 to P8, and P10 to P15 were amplified from the genomic DNA of cv. Cuiguan and inserted into the pGreenII 0800-LUC vector as reporters. Additionally, the full-length coding sequences of *PbHB.G7.2*, *PbHB.G1*, and *PbHB.G2.1* were inserted into the pSAK277 vector as effectors (35S::PbHB.G7.2, 35S::PbHB.G1, and 35S::PbHB.G2.1). The transformation of constructs and *Agrobacterium*-introduced infiltration were identical to a previous report [50]. Luminescence assay and the measurement of Firefly luciferase (Luc) and Renilla luciferase (Ren) activities were identical to a previous report [50]. At least six transient assay measurements were contained for each assay. All of the primer sequences are listed in Table S4 (see online supplementary material).

### Yeast-one-hybrid assay (Y1H)

The full-length coding sequences of *PbHB.G7.2*, *PbHB.G1*, and *PbHB.G2.1* were individually inserted into the pGADT7 vector. The 2000-bp sequences upstream of *PbACS1b* and the P9, P16, and P17 sequences were individually inserted into the pAbAi vector. The Y1H assay was performed using the Matchmaker Gold Yeast One-Hybrid Library Screening System (Clontech, Palo Alto, CA, USA). All of the primer sequences are listed in Table S4 (see online supplementary material).

### Electrophoretic mobility shift assay (EMSA)

The full-length coding sequences of *PbHB.G7.2*, *PbHB.G1*, and *PbHB.G2.1* were inserted into the pCold-TF expression vector and then introduced to *E. coli* strain BL21. Extraction and purification of the recombinant proteins were identical to a previous study [35]. Moreover, based on the results detected by the dual-luciferase and Y1H assays, the sequences of the biotin-labeled probes were 5′-CTTATGGGTCGGTTTTTCTACATTTGAATTTTGA-3′ in the P9 sequences, 5′-TTATATGTTGTATCTTATTTCATCGTTGATTAC-3′ in the P16 sequences, and 5′-CCTCAAGGAAATAGTAATACCCTTTAGATTTGAAGTCCTA-3′ in the P17 sequences. An EMSA was performed according to a previous study [35]. All of the primer sequences are listed in Table S4 (see online supplementary material).

### Yeast-two-hybrid assay (Y2H)

A cDNA library was constructed with the mRNA extracted from the ripening fruits of cv. Cuiguan using Make Your Own ‘Mate & Plate’ Library System (Clontech). The Y2H screening was performed using the Matchmaker Gold Yeast Two-Hybrid System (Clontech). The full-length coding sequences of *PbHB.G.7.2* were inserted into the pGBKT7 vector, and then used as bait for Y2H screening.

To identify the PbHB.G7.2-PbHB.G1, and PbHB.G7.2-PbHB.G2.1 interaction regions in the three proteins, the full-length sequences of *PbHB.G1* and *PbHB.G2.1* and the fragments *PbHB.G1-N* (1–181 aa), *PbHB.G1-D* (182–272 aa), *PbHB.G1-C* (273–345 aa), *PbHB.G2.1-N* (1–194 aa), *PbHB.G2.1-D* (195–285 aa), and *PbHB.G2.1-C* (286–369 aa) were inserted into the pGADT7 vector. Meanwhile, the fragments *PbHB.G7.2-N* (1–143 aa), *PbHB.G7.2-D* (144–235 aa), *PbHB.G7.2-C* (236–304 aa), *PbHB.G7.2-(N + D)* (1–235 aa), and *PbHB.G7.2-(D + C)* (144–304 aa) were inserted into the pGBKT7 vector. The Y2H was conducted in the yeast strain Y2HGold using the Matchmaker Gold Yeast Two-Hybrid System (Clontech). All of the primer sequences are listed in Table S4 (see online supplementary material).

### Firefly luciferase complementation imaging (FLCI) assay

The full-length sequences of *PbHB.G7.2* and the fragments *PbHB.G7.2-(D + C)* were inserted into the polyclonal sites of the pCAMBIA1300-nLuc vector. The fragments *PbHB.G1-D* and *PbHB.G2.1-D* and the full-length coding sequences of *PbHB.G1* and *PbHB.G2.1* were inserted into the pCAMBIA1300-cLuc vector. These constructs were assigned as four groups as PbHB.G7.2-nLuc/cLuc-PbHB.G2.1, PbHB.G7.2-nLuc/cLuc-PbHB.G1, PbHB.G7.2-(D + C)-nLuc/cLuc-PbHB.G2.1-D, and PbHB.G7.2-(D + C)-nLuc/cLuc-PbHB.G1-D. After introducing these constructs into *A. tumefaciens* strain EHA105, the constructs in each group were co-transformed into tobacco leaves. All incubations and conditions of transient expression were identical to a previous report [51]. Luciferase activity was checked at 3 days after injection (DAI) using the Luminescence & Fluorescence Imaging System (PIXIS 1024B/BUV, Teledyne Princeton Instruments, Princeton, NJ, USA). All of the primer sequences are listed in Table S4 (see online supplementary material).

### In vivo co-immunoprecipitation (Co-IP) assay

The full-length coding sequences of *PbHB.G7.2* and the fragments *PbHB.G7.2-(D + C)* were individually inserted into the pEarleyGate201-YN vector to ligate with HA tag. The full-length sequences of *PbHB.G1*, *PbHB.G2.1* and the fragments *PbHB.G1-D* and *PbHB.G2.1-D* were individually inserted into the pEarleyGate202-YC vector to ligate with Flag tag. These constructs were introduced into *A. tumefaciens* strain GV3101 and coinfiltrated into tobacco leaves as follows: PbHB.G7.2-HA/PbHB.G1-Flag, PbHB.G7.2-HA/PbHB.G2.1-Flag, PbHB.G7.2-(D + C)-HA/PbHB.G1-D-Flag, and PbHB.G7.2-(D + C)-HA/PbHB.G2.1-D-Flag. Co-IP assay was performed according to a previous study [12]. Anti-HA and anti-Flag antibodies were provided by the Abmart Company (Shanghai, China). All of the primer sequences are listed in Table S4 (see online supplementary material).

### Quantitative real-time PCR and statistical analysis

Quantitative real-time PCR (qRT-PCR) was performed as described in a previous study [32]. All of the primer sequences are listed in Table S4 (see online supplementary material). Means and standard errors were calculated using ANOVA. The significance at *P* < 0.05 was displayed by the lowercase letters (such as a, b, and c) or asterisk.

## Supplementary Material

Web_Material_uhae086

## Data Availability

The data for this article are available in the article or in its supplementary material.
