# Supplementary material for: Interaction among homeodomain transcription factors mediates ethylene biosynthesis during pear fruit ripening
Source: Hortic Res. 2024 Mar 28;11(5):uhae086. doi: 10.1093/hr/uhae086 (PMC11116900; doi:10.1093/hr/uhae086)
Supplement: Web_Material_uhae086 [file web_material_uhae086.zip › Supplementary figures.docx]

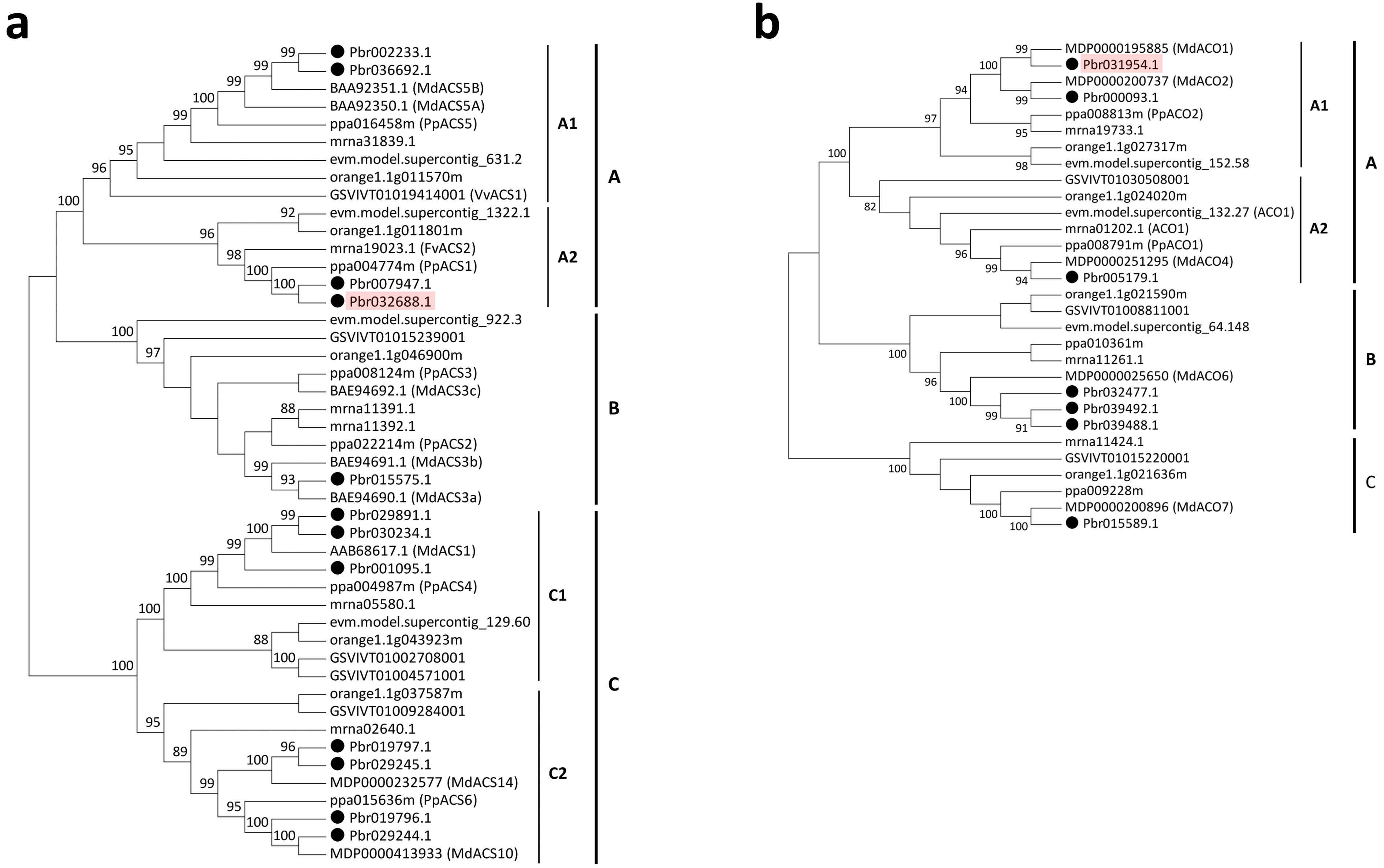


**Figure S1.** Phylogenetic analysis of *ACS* and *ACO* genes in pear, peach, apple, strawberry, papaya, orange, and orange. (a) The *ACS* genes were catergorized into three groups: A, B, and C. Among these groups, both A and C consisted of two sub-groups. *Pbr032688.1* clustered with *PpACS1* in sub-group A2, and was therefore designated as *PbACS1b*. (b) The *ACO* genes were also classified into three groups: A, B, and C. Group A consisted of sub-groups A1 and A2. *Pbr31954.1* clustered with *MdACO1* in sub-group A1, and was designated as *PbACO1*.


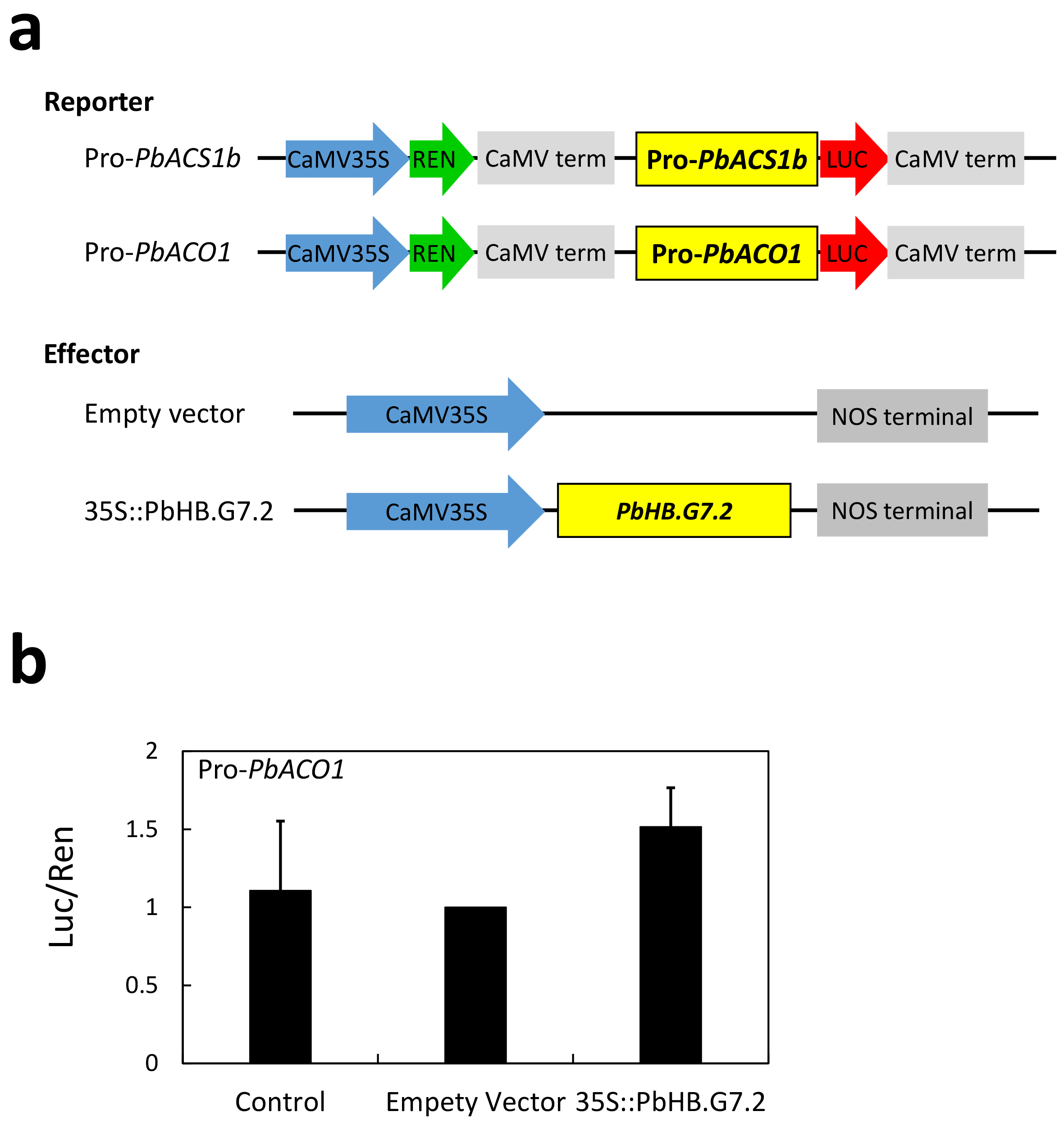


**Figure S2**. PbHB.G7.2 does not affect the activity of the *PbACO1* promoter. (a) Reporters and effectors were constructed for the dual-luciferase assay. (b) The dual-luciferase assay demonstrated that the *LUC* activity driven by the *PbACO1* promoter in the leaves infiltrated with 35S::PbHB.G7.2 was comparable to that in the leaves infiltrated with empty vector. Mean values and standard errors were calculated using ANOVA.


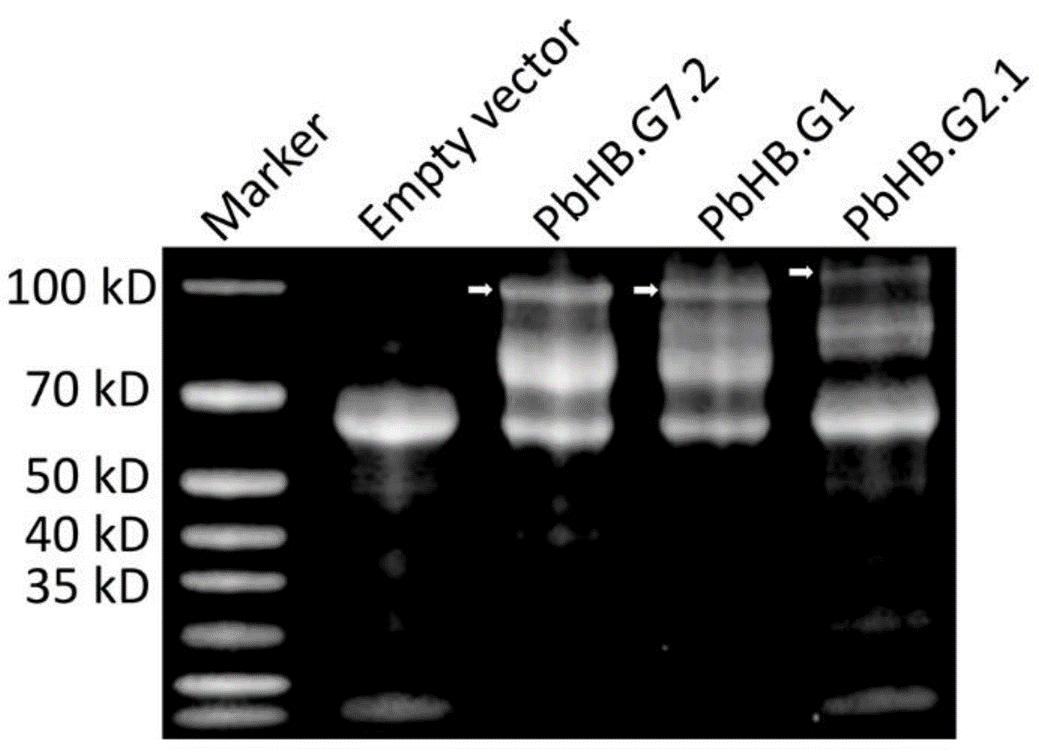


**Figure S3.** The recombinant HB proteins were extracted and purified from *E.coli*. The purified proteins were separated using SDS-PAGE gel electrophoresis and subsequently stained with Coomassie Blue. The Coomassie-stained gel image was captured using ChemiDoc™ MP Imaging System (Bio-Rad) with Far Red Epi illumination at and exposure time of 21.27 seconds. Arrow with white color indicates the target protein.


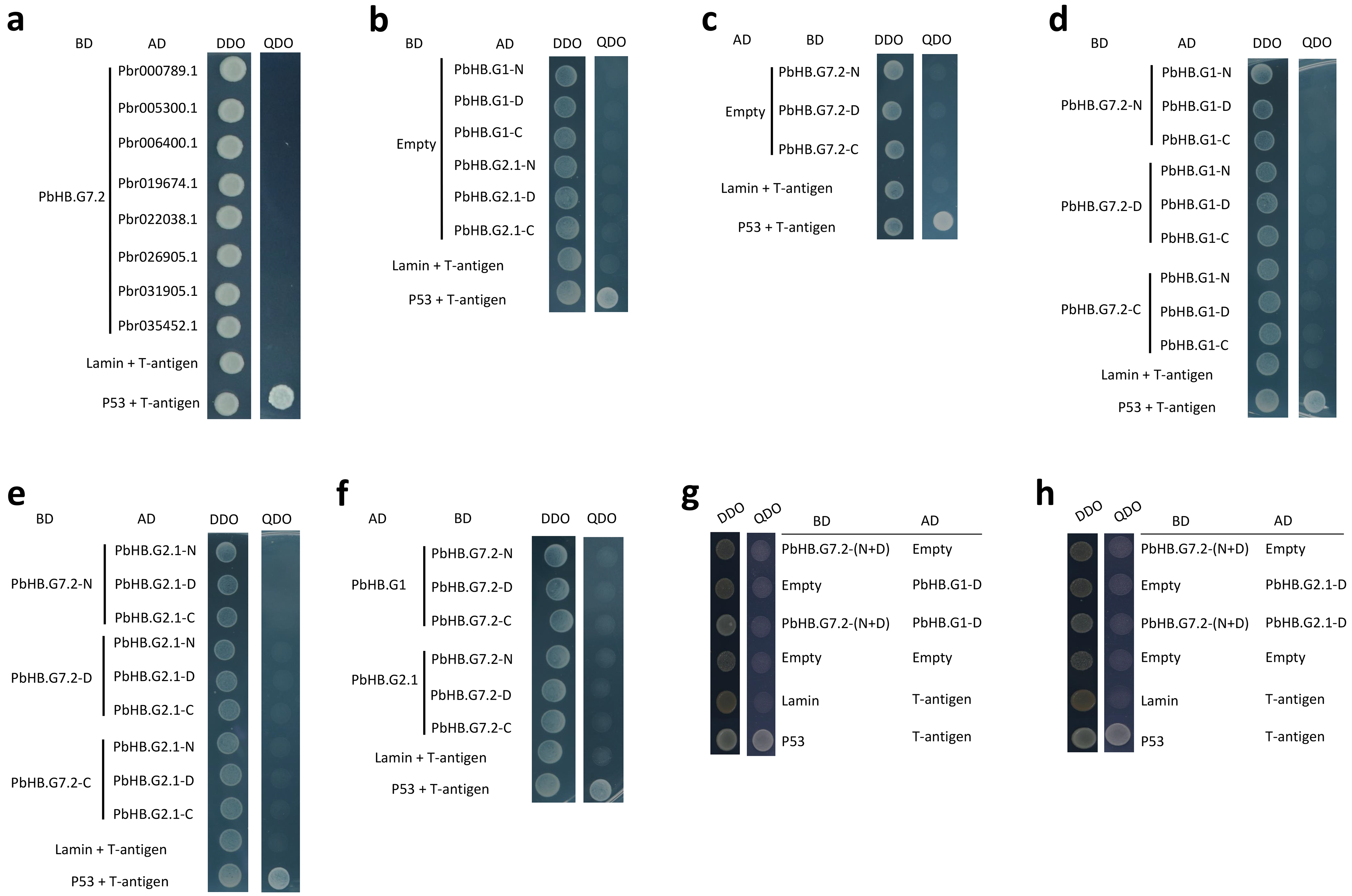


**Figure S4.** Y2H assay was conducted to investigate the interaction of PbHB.G7.2 with PbHB.G1 and PbHB.G2.1. (a) No interaction was observed between PbHB.G7.2 and any of the tested proteins. (b) PbHB.G1-N, PbHB.G1-D, PbHB.G1-C, PbHB.G2.1-N, PbHB.G2.1-D, and PbHB.G2.1-C did not exhibit self-activation in yeast cells. (c) PbHB.G7.2-N, PbHB.G7.2-D, and PbHB.G7.2-C did not exhibit self-activation in yeast cells. (d) No interaction was detected between any two fragments of PbHB.G7.2 and PbHB.G1. (e) No interaction was detected between any two fragments of PbHB.G7.2 and PbHB.G2.1. (f) No interaction was detected between any fragment of PbHB.G7.2 and the whole proteins of PbHB.G1 and PbHB.G2.1. (g) No interaction was detected between PbHB.G7.2-(N+D) and PbHB.G1-D. (h) No interaction was detected between PbHB.G7.2-(N+D) and PbHB.G2.1-D. DDO, SD medium lacking Trp and Leu; QDO, SD medium lacking Trp, Leu, His, and Ade; X-a-gal, QDO medium containing x-a-gal and AbA. The positive control (PC) consisted of P53 and T-antigen. Two negative controls (NC) were involved in each of Y2H assays. One control untilized AD and BD vectors (Empty), and the other control employed Lamin and T-antigen. Blue plaques indicate protein-protein interactions.


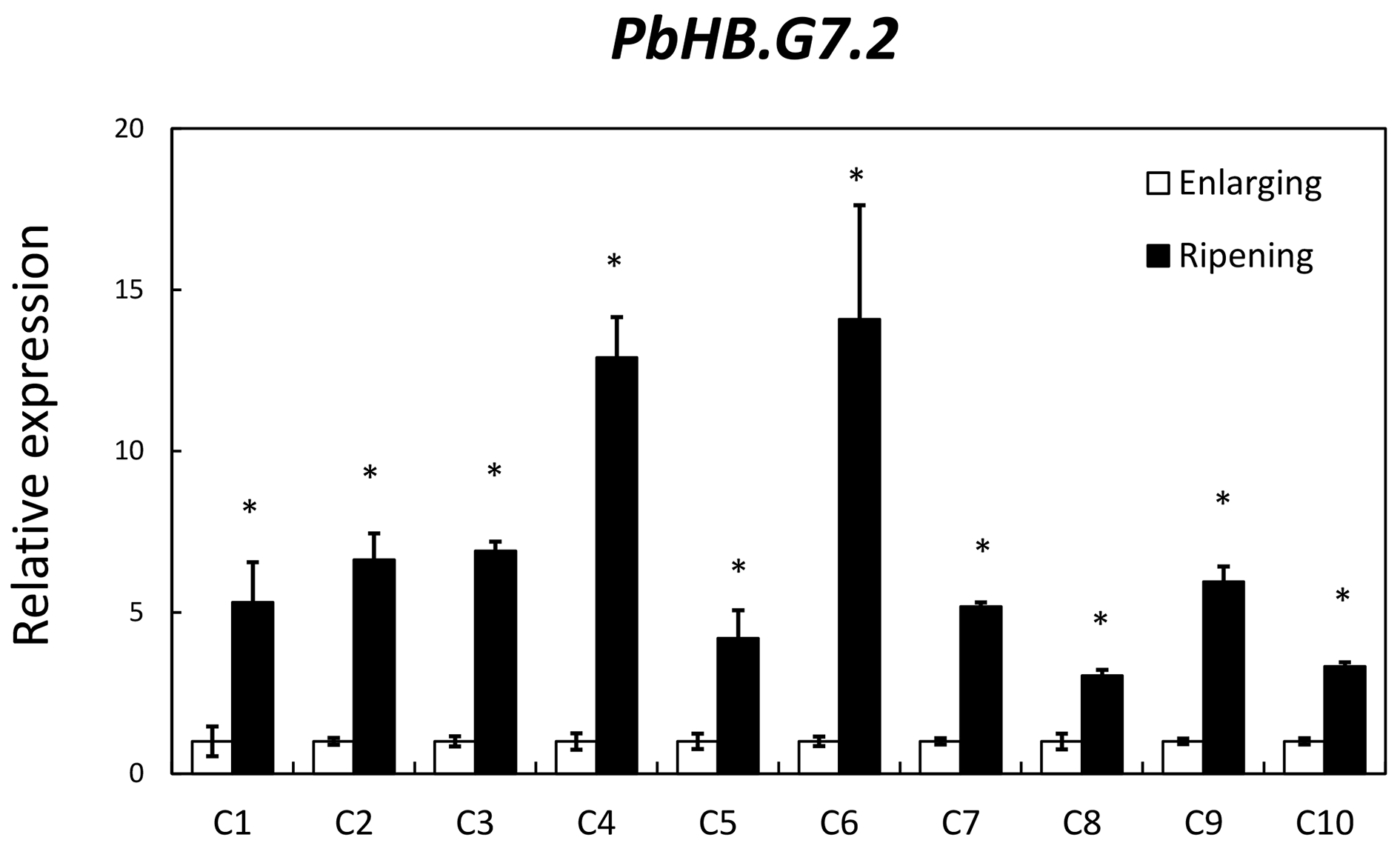


**Figure S5.** The expression patterns of *PbHB.G7.2* were analyzed in enlarging and ripening fruits of 10 different pear cultivars. The cultivars were labeled as C1 (Huasu), C2 (Zhongli No.1), C3 (Xizilv), C4 (Zaomeisu), C5 (Liuyueshuang), C6 (Jinhua), C7 (Eli No.1), C8 (Ningmenghuang), C9 (Jinsui No.1), and C10 (Xinhang). Mean values and standard errors were calculated using ANOVA. Asterisk indicate the level of significance at *P* < 0.05.


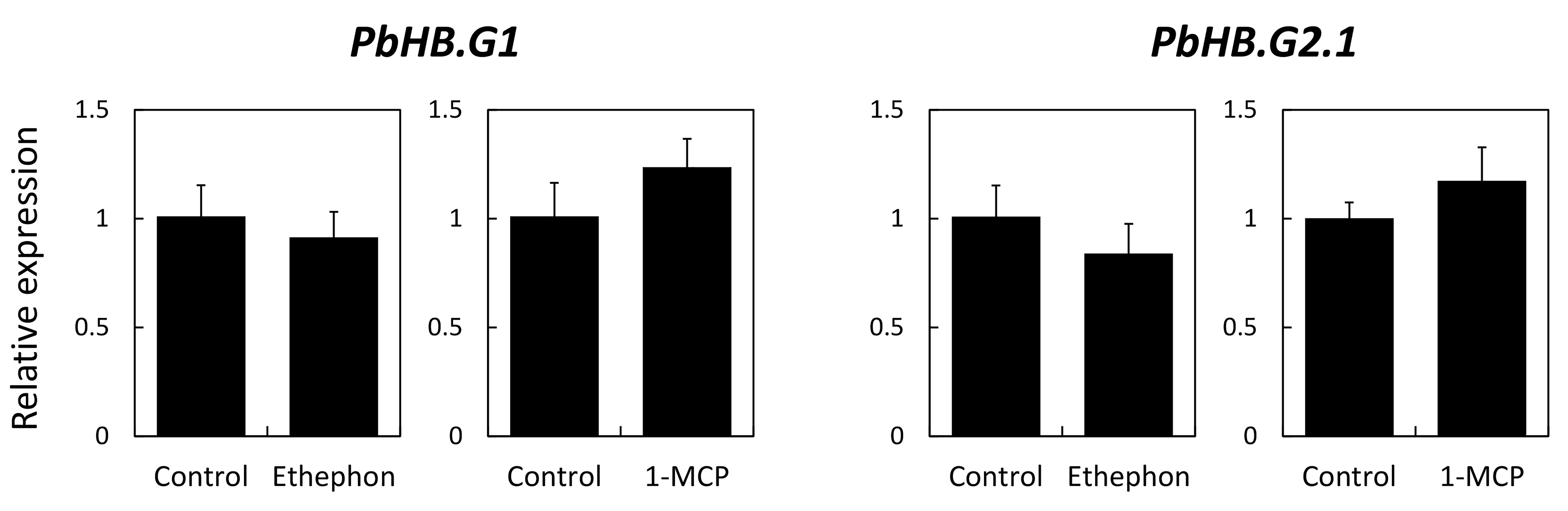


**Figure S6.** The expression levels of *PbHB.G1*, and *PbHB.G2.1* were unaffected by ethylene signaling. The fruits with ethephon and 1-MCP treatments exhibited similar expression levels of *PbHB.G1* and *PbHB.G2.1* compared to the untreated control fruits. Mean values and standard errors were calculated using ANOVA.
